# Long-Range and High-Efficiency Plasmon-Assisted Förster Resonance Energy Transfer

**DOI:** 10.1021/acs.jpcc.3c04281

**Published:** 2023-10-27

**Authors:** Abdullah O. Hamza, Ali Al-Dulaimi, Jean-Sebastien G. Bouillard, Ali M. Adawi

**Affiliations:** † Department of Physics, University of Hull, Cottingham Road, Hull HU6 7RX, U.K.; ‡ G. W. Gray Centre for Advanced Materials, University of Hull, Cottingham Road, Hull HU6 7RX, U.K.; § Department of Physics, College of Science, Salahaddin University-Erbil, Erbil 44002, Kurdistan Region, Iraq

## Abstract

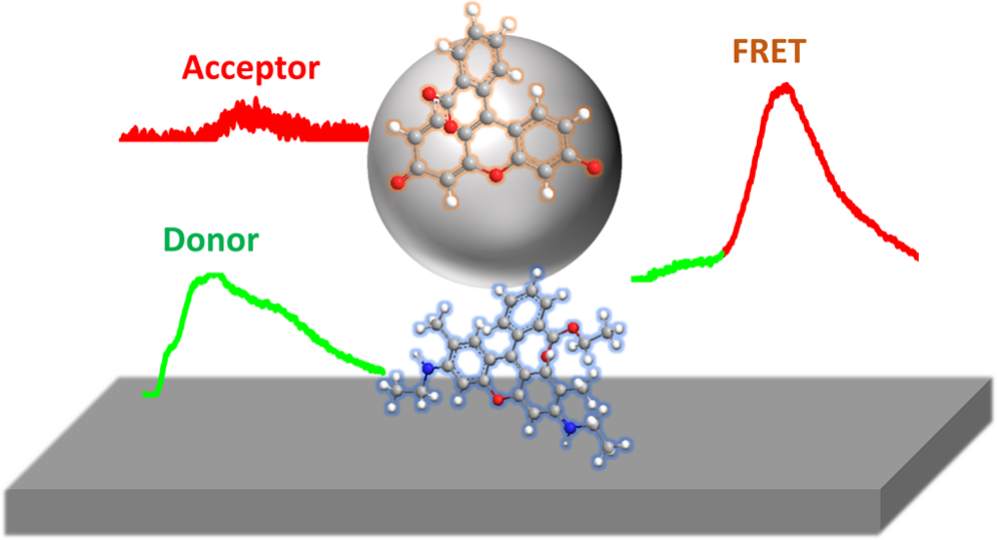

The development of a long-range and efficient Förster resonance energy transfer (FRET) process is essential for its application in key enabling optoelectronic and sensing technologies. Via controlling the delocalization of the donor’s electric field and Purcell enhancements, we experimentally demonstrate long-range and high-efficiency Förster resonance energy transfer using a plasmonic nanogap formed between a silver nanoparticle and an extended silver film. Our measurements show that the FRET range can be extended to over 200 nm while keeping the FRET efficiency over 0.38, achieving an efficiency enhancement factor of ∼10^8^ with respect to a homogeneous environment. Reducing Purcell enhancements by removing the extended silver film increases the FRET efficiency to 0.55, at the expense of the FRET rate. We support our experimental findings with numerical calculations based on three-dimensional finite difference time-domain calculations and treat the donor and acceptor as classical dipoles. Our enhanced FRET range and efficiency structures provide a powerful strategy to develop novel optoelectronic devices and long-range FRET imaging and sensing systems.

## 1 Introduction

Förster resonance energy transfer (FRET) is a short-range phenomenon with a wide range of applications in physics, chemistry, and biological processes at the molecular level.^1−3^ FRET also plays an important role in developing novel strategies to enhance the functionality and the efficiency of a wide range of applications such as light-harvesting systems,^4^ optical networks,^5,6^ color-tuning LED,^7,8^ and sensing.^9,10^ However, the short-range nature of FRET^11^ (∼10 nm) drastically hinders its use in such key enabling technologies. It is therefore critical to develop strategies to precisely control the FRET rate, efficiency, and range.

The FRET process is affected by the local electromagnetic field in the vicinity of the donor–acceptor pair.^12,13^ More precisely, the FRET rate^12,14^ is proportional to the square of the donor field, *E*_D_, at the location of the acceptor, *r*_A_, (Γ_FRET_ ∝ |*E*_D_ (*r*_A_)|^2^). Physically, this means that the FRET rate, Γ_FRET_, efficiency, and range all depend on the delocalized nature of the donor’s electric field at the location of the acceptor,^15^ a quantity that can be carefully controlled via engineering the electromagnetic environment surrounding the donor–acceptor pair to modify the FRET process.^15−24^

Several studies investigated the modification of the FRET range by coupling to aluminum waveguides,^25,26^ across^16,27^ or along^1,28^ metallic films, across a silver microcavity,^29^ arrays of metallic particles,^30−32^ hyperbolic metamaterials,^33−35^ plasmonic nanoparticles,^36,37^ surface lattice resonances,^15,23,38^ or plasmonic nanorods.^22,39^ Using such plasmonic systems, FRET ranges of up to 7 μm have been reported.^28^ However, despite successful progress in extending the FRET range to the micrometer scale, retaining an efficient FRET process over long ranges remains a challenge. This is in part due to the Purcell enhancement and its role in modifying the donor’s radiative and nonradiative decay rates in the presence of plasmonic structures.^25,27,29^ For example, Golmakaniyoon et al.^27^ reported a FRET efficiency of 0.26 and a FRET range of 130 nm using stratified metal-dielectric nanostructures. Baibakov et al.^25^ reported 0.05 FRET efficiency over 150 nm FRET range on the single-molecule level using zero-mode waveguide nanoapertures.

Boddeti et al.^15^ demonstrated a FRET range of up to 800 nm combined with a FRET efficiency of 0.05 using surface lattice resonances in a plasmonic nanoparticle array. Here, it is worth mentioning that in this system, a FRET efficiency of ∼0.35 was achieved for the 4 nm ≤ FRET range ≤100 nm. Higgins et al.^32^ achieved a plasmon-mediated energy transfer efficiency of up to ∼51% between a layer of quantum dots and InGaN quantum well separated by a 40 nm ordered silver nanoring array. Via coupling to surface plasmons on single-crystalline silver nanowires, De Torres et al.^22^ demonstrated a FRET range of 1.3 μm with a FRET efficiency of 0.025. Bouchet et al.^28^ showed that surface plasmon polaritons can enhance the FRET range up to 7 μm with a theoretically estimated FRET efficiency of <10^–6^, linked to a plasmon-assisted energy transfer efficiency enhancement factor of 30 with respect to free space.

**Figure 1:**
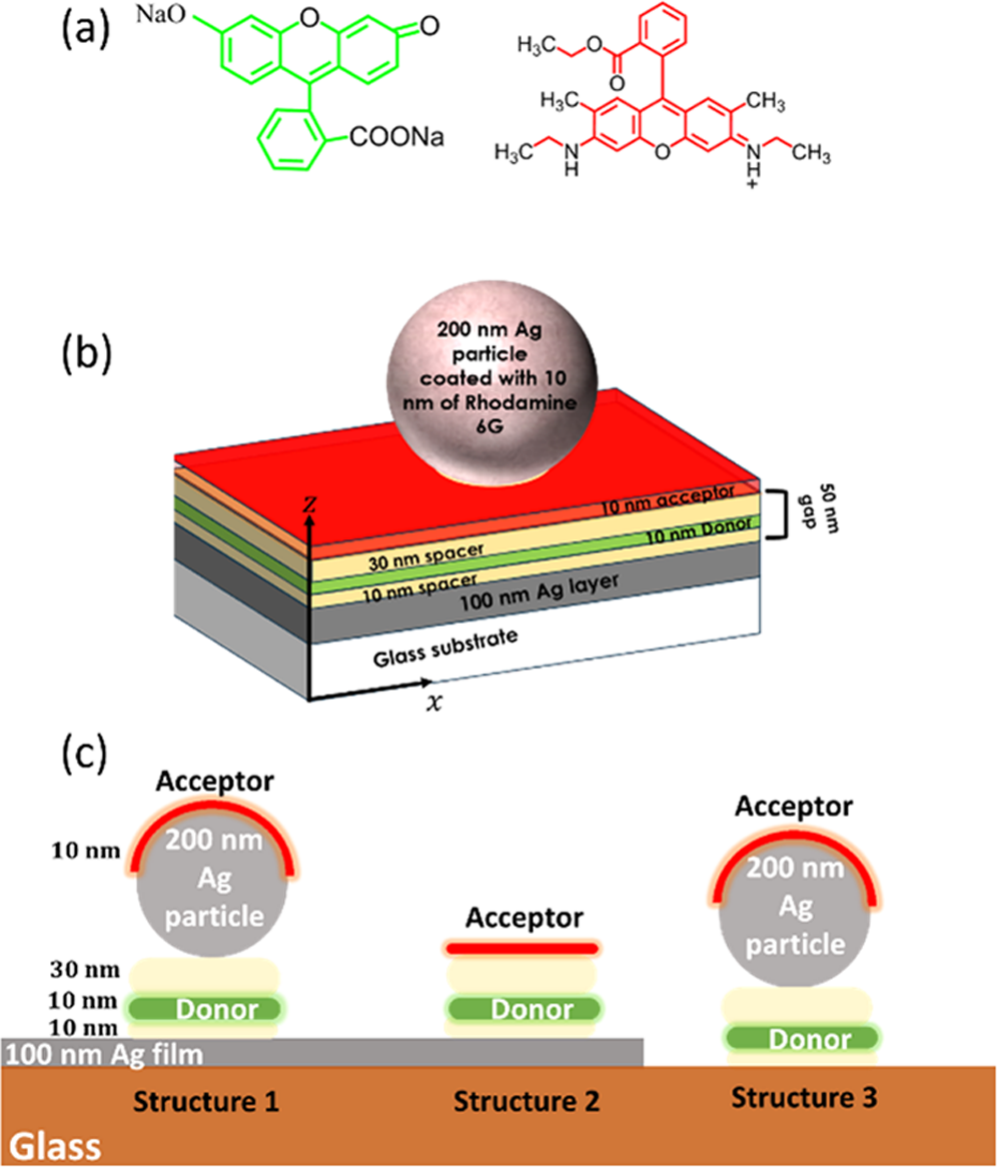
(a) Chemical structure of the donor–acceptor molecules (disodium fluorescein–rhodamine 6G) used in this work. (b, c) Schematic of the studied structures.

In this work, we investigate the FRET range and efficiency using a 50 nm plasmonic nanogap consisting of a 200 nm diameter silver particle coupled to an extended silver film (see Figure 1b,c). In this system, the distance between the donor and the acceptor is 230 nm , corresponding to the intermediate dipole–dipole separation of the near-field regime.^14,40,41^ We found that using a plasmonic nanogap, the FRET range can be extended to over 200 nm while keeping the FRET efficiency over 0.38, providing an efficiency enhancement factor of ∼10^8^ with respect to the homogeneous environment. By reducing Purcell enhancements via removing the extended silver film, the FRET efficiency can be increased by a factor of 1.4 combined with a reduction in the FRET rate by a factor of 1.25. These findings pave the way toward designing and producing long-range high-efficiency FRET structures via controlling the delocalization of the donor’s electric field and Purcell enhancements in the structure.

## 2 Materials and Methods

### 2.1 Nanogap Fabrication

The investigated plasmonic structures were fabricated on a 20 mm × 15 mm glass substrate coated with a 100 nm-thick silver layer deposited by thermal evaporation through a mask containing six 8 mm × 1.5 mm rectangles. This allows for the simultaneous fabrication of the nanogap structure and reference geometries on the same sample. This was followed by a 10 nm spacing layer of Zeonex (Zeon Chemicals Europe Ltd.) deposited by spin-coating a 3 mg·mL^–1^ solution of Zeonex in toluene at a speed of 2000 rpm for 30 s. A 10 nm donor layer consisting of PMA (poly(methacrylic acid), Scientific Polymer Products Inc.) doped with the donor molecule, disodium fluorescein (see Figure 1a), at a concentration of 5% in weight was created by spin-coating a 3 mg·mL^–1^ solution of doped PMA in ethanol at a speed of 2000 rpm for 30 s. A final 30 nm spacing layer of Zeonex was then deposited by spin-coating a 9 mg·mL^–1^ solution of Zeonex in toluene at a speed of 2000 rpm for 30 s. AFM measurements for the surface of the Zeonex layer (Figure S1) indicate that the average surface roughness of the structure is 1.5 nm. Here, it is worth mentioning that the Zeonex layer is cross-soluble with the PMA layer.^20^ To complete the structure, 200 nm silver nanoparticles (nanoComposix) suspended in ethanol at a concentration of ∼2 × 10^–4^ g·L^–1^ were spin-coated onto the Zeonex surface at a speed of 2000 rpm for 30 s, providing a spacing of several micrometers between the nanoparticles.^42,43^ Finally, a 10 nm acceptor layer consisting of a PMA layer doped with rhodamine 6G (see Figure 1a) as the acceptor molecule at a concentration of 0.5% in weight was deposited by spin coating. A Bruker Dektak XT profilometer was used to measure the thickness of all the used layers (see Figures S1–S3).

**Figure 2:**
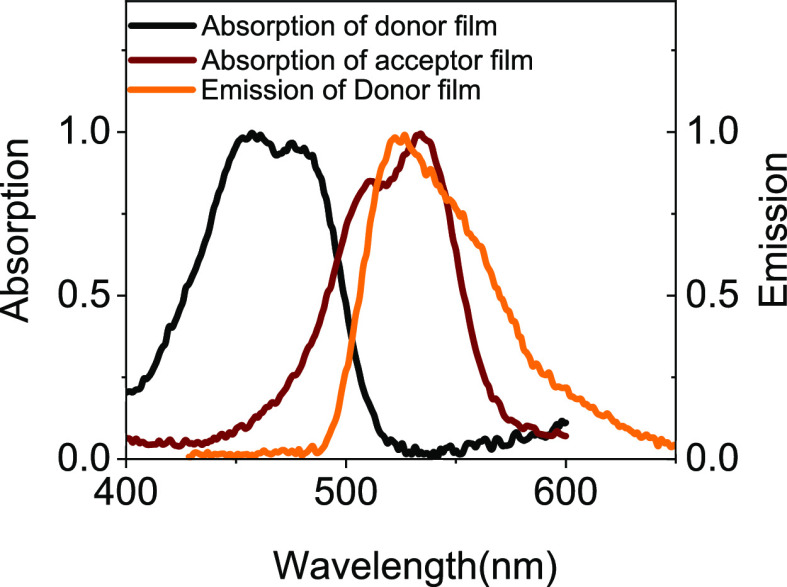
Normalized absorption and emission spectral of the donor disodium fluorescein film on glass alongside the absorption spectrum of the acceptor rhodamine 6G film on glass. The donor emission spectrum was measured using 405 nm excitation wavelength.

The final structures are schematically presented in Figure 1b,c. Here, it is important to stress that we start with a donor–acceptor separation of 230 nm, much larger than the Förster energy transfer range in the homogeneous environment. For our system, the theoretical FRET efficiency without the plasmonic nanostructures (in the homogeneous environment) can be calculated using , where *R*_0_ is the Förster radius and *R* is the distance between the donor and acceptor. For our system,^20^*R*_0_ = 9.3 nm, resulting in expected FRET efficiencies in homogeneous environments of the order of 10^–8^. The donor–acceptor (disodium fluorescein–rhodamine 6G) pair was chosen due to the large spectral overlap between the disodium fluorescein emission spectrum and rhodamine 6G absorption spectrum (see Figure 2), along with the large spectral overlap between these spectra and the plasmonic resonances of both spherical silver nanoparticles^44^ and silver-based plasmonic nanogaps.^42,45^

The nanogap width was set to 50 nm to obtain increased FRET efficiency at longer ranges, by providing the required donor field delocalization combined with minimized Purcell enhancements.^23,46^

### 2.2 Time-Resolved Measurements and Analysis

For the time-resolved fluorescence measurements, the donor was excited using a 405 nm pulsed diode laser with 40 ps pulse width and 80 MHz repetition rate. The excitation laser was focused on the sample using a 100× Mitutoyo infinity-corrected objective lens with numerical aperture NA = 0.7. The same objective was used to collect the donor emission signal, which was then directed toward an iHR320 Horiba spectrometer where it was dispersed using a 150 line/mm grating onto an HPM-100 time-correlated single-photon counter. The detection wavelength was set at the donor emission wavelength λ_donor_ = 516 nm with a bandwidth of 37.5 nm. The donor emission lifetime for the various structures was determined via the deconvolution of the instrument response function (IRF) and then fitted to a double-exponential model: . The fast decay component *t*_1_ is attributed to molecules coupled to the plasmonic nanostructure/plasmonic nanostructure–acceptor systems^47−49^ and was used to calculate the different rates (Γ_T_, Γ_D_ and Γ_D_0__) using . Each point in the Purcell factor *F*_p_, FRET rate , and FRET efficiency η corresponds to the average of measurements from five identical structures.

### 2.3 3D FDTD Calculations

3D FDTD calculations (Ansys Lumerical software) were used to calculate the normalized FRET rate *G*_FRET_ using the approach developed in ref (20) and (24). In our calculations, we treated the donor and the acceptor as classical dipoles pointing along the *z*-direction (perpendicular to the metallic film and see Figure 1b), with the donor located at either *x* = 0, 25, 50, 75, and 100 nm in the plane of the donor layer (see Figure 1b). The acceptor layer was modeled as a 10 nm shell with a refractive index of 1.43 surrounding the silver particle down to the contact points with the Zeonex layer. In the calculations, we applied 1 nm uniform grid spacing and stretched coordinate perfectly matching layer boundary conditions. Calculations were terminated when the electric field reached 10^–5^ of its original value. The dielectric function of silver was described using the experimental data from Johnson and Christy.^50^

## 3 Results and Discussion

**Figure 3:**
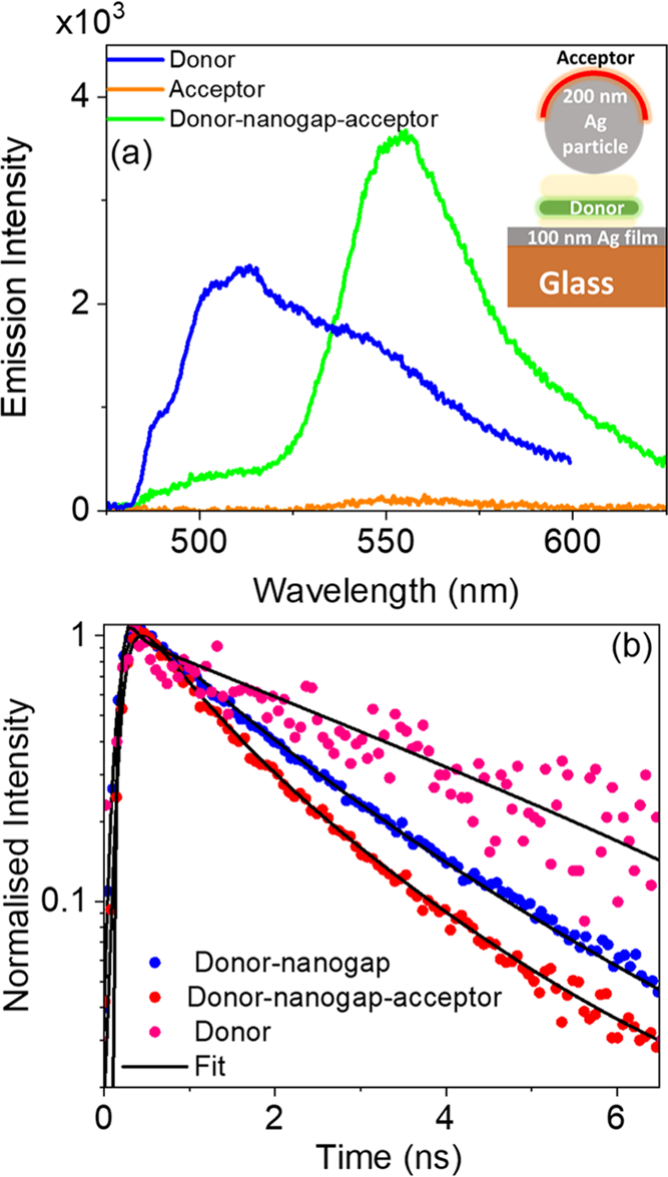
(a) Measured fluorescence spectrum of the donor–nanogap–acceptor structure of 50 nm gap width and a silver particle of 200 nm diameter, alongside the emission spectra of the donor film on glass and acceptor film on glass. (b) Fluorescence decay curves of donor–nanogap and donor–nanogap–acceptor measured from a 50 nm plasmonic nanogap with a silver particle of 200 nm diameter. For comparison, data are shown for the donor film on glass.

In the first instance, to characterize our donor–acceptor pair, the CW fluorescence of the donor–nanogap–acceptor system (inset of Figure 3a) was measured, along with reference films of the isolated donor and isolated acceptor on glass (Figure 3a). In those measurements, a 405 nm diode laser was used to selectively excite the donor (disodium fluorescein) but not the acceptor (rhodamine 6G) molecules (Figure 2), a choice further validated by the low fluorescence emission of the isolated acceptor film when compared to the isolated donor film. Conversely, in the donor–nanogap–acceptor structure, we see strong quenching of the donor emission intensity combined with a strong enhancement in the acceptor’s fluorescence (Figure 3a), clearly demonstrating that FRET is taking place across the plasmonic nanogap.

To confirm the FRET process and to quantify the FRET rate and efficiency, the emission rate of the donor has been investigated in the donor–nanogap–acceptor (structure 1). In parallel, two different reference samples were considered: (i) the donor on glass allowing us to determine the spontaneous emission rate of the donor in free space (Γ_D_0__) and (ii) the donor only in the nanogap providing the emission rate of the donor in the presence of the nanogap (Γ_D_), which can be linked to the Purcell enhancement by  Purcell enhancement (see Figure 3b).

From the decay rate measurements of the donor, it is observed that the nanogap provides a Purcell enhancement of 2.5 times due to the modification of the local density of optical states (LDOS) in the nanogap (see Figure 3b). The presence of the acceptor, in the donor–nanogap–acceptor structure, further enhances the donor emission rate, evidencing the FRET process across the nanogap, resulting in an extension of the FRET range from^20^ 9.3 nm to over 200 nm. This drastic increase in the FRET range can be attributed to the strong delocalization of the donor’s electric field in the presence of the plasmonic nanogap.

**Figure 4:**
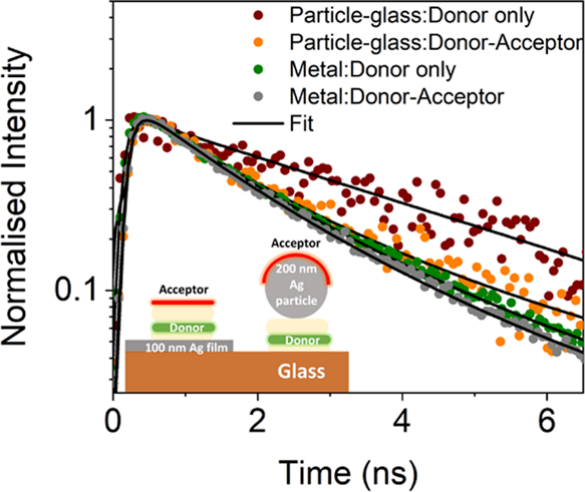
Measured fluorescence decay curves of glass–donor–200 nm silver particle, glass–donor–acceptor–200 nm silver particle, and metal–donor and metal–donor–acceptor structures.

For comparison, we also measured the donor decay rate from the metal film alone and the nanoparticle alone (structures 2 and 3, respectively), both with and without the acceptor molecules (see Figure 1c and the inset in Figure 4). From the results in Figure 4, it is apparent that FRET is also taking place across an isolated 200 nm silver particle without the presence of the underlying metallic film, which supports our earlier results in Figure 3.

To quantify the FRET rate, the total decay rate of the donor (Γ_T_) can be written as the sum of the rates corresponding to the two different decay processes in the presence of the structure: the FRET rate (Γ_FRET_) and the spontaneous emission rate of the donor (Γ_D_), thus
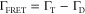
1

**Figure 5:**
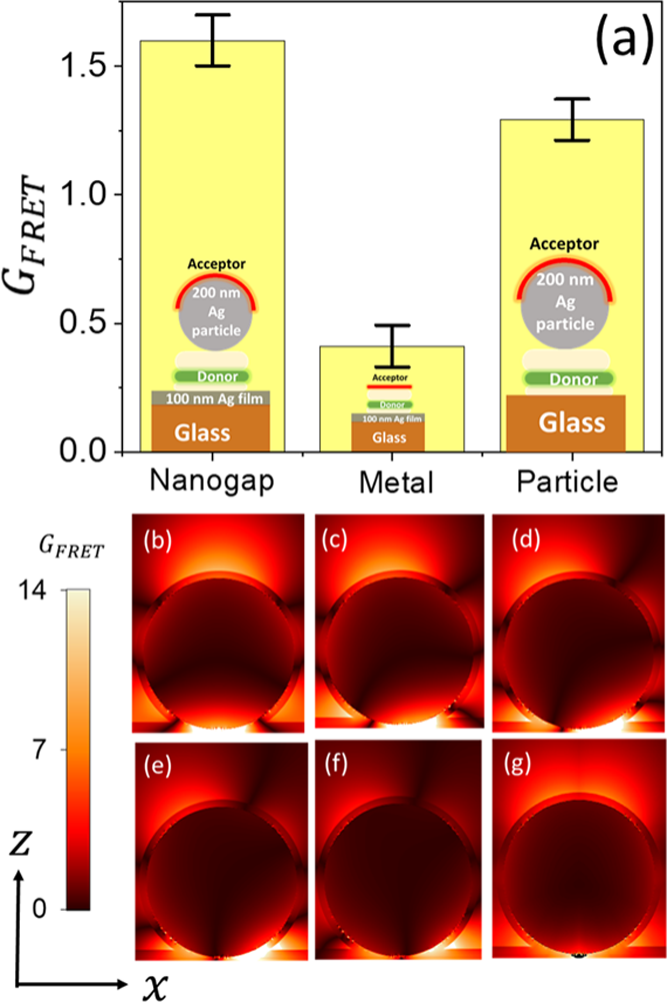
(a) Measured normalized FRET rate  for donor–nanogap–acceptor structure of 50 nm gap width and a silver particle of 200 nm diameter, glass–donor–acceptor–200 nm silver particle, and metal–donor–acceptor structures. (b–f) Calculated normalized FRET rate  as a function of the donor location in the plane of the nanogap (*x* = 0, 25, 50, 75, and 100 nm) at the donor emission wavelength λ_donor_ = 516 nm. (g) *G*_FRET_ map averaged over the donor location.

Figure 5a shows the FRET rate normalized to the donor film on glass  for the different structures. From these results, it is observed that both the plasmonic nanogap and the isolated plasmonic nanoparticle (structures 1 and 3, respectively) assist the interaction between the donor and acceptor molecules and extend the FRET range beyond its typical range from 10 to over 200 nm. In addition to this large extension of the FRET range, the FRET rate associated with the plasmonic nanogap is enhanced by a factor of 1.25 relative to that of an isolated silver particle. In parallel, with the metal film alone, in the absence of the metallic nanoparticle, the donor emission decay rate is mainly dominated by Purcell enhancement rather than FRET (see Figures 4 and 5a).

To confirm our observed long-range FRET process, we numerically calculated the normalized FRET rate  as a function of the donor location in the plane of the nanogap and acceptor position in the *x*–*z* plane at the donor emission wavelength λ_donor_ = 516 nm (Figure 5b–g). It can be seen that FRET takes place across the plasmonic nanogap, in support of our experimental observation in Figure 3, independently of the donor location in the plane of the nanogap. Also, our results are in accordance with previous theoretical studies.^36,37^ Although the location of the donor modifies the *G*_FRET_ spatial variation (Figure 5b–f), the change in the normalized FRET rate is limited. Additionally, averaging the *G*_FRET_ over the donor position, mimicking the experimental molecular film, results in the maximum *G*_FRET_ located on the upper hemisphere of the nanoparticle forming the nanogap (Figure 5g). Furthermore, the value of the spatially averaged *G*_FRET_ over the top hemisphere of the particle, within the acceptor layer, is 2.8, which is in line with the experimentally measured value of 1.6, further supporting our experimental findings.

In parallel to the FRET rate, the FRET efficiency is a measure of the likelihood of the excited donor to undergo the FRET process and is defined as
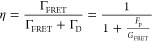
2By rewriting the FRET efficiency in this form, the competition between the Purcell enhancement and FRET process becomes apparent, and consequently, achieving high FRET efficiency η requires *F*_p_ ≪ *G*_FRET_.

**Figure 6:**
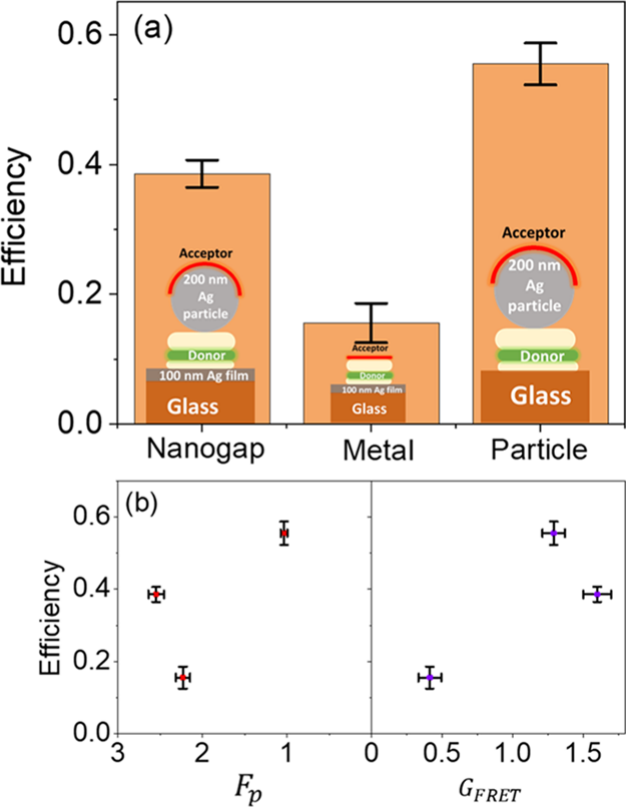
(a) FRET efficiency η for donor–nanogap–acceptor structure of 50 nm gap width and a silver particle of 200 nm diameter, glass–donor–acceptor–200 nm silver particle, and metal–donor–acceptor structures. (b) Dependence of the FRET efficiency on the Purcell enhancement  and the normalized FRET rate .

In Figure 6, we plot the FRET efficiency η for the different investigated structures. The efficiency of the nanogap structure is 0.38, almost 8 orders of magnitude efficiency enhancement factor with respect to donor–acceptor molecules placed in the homogeneous environment and separated by a distance of 230 nm. In comparison, the FRET efficiency assisted by a single silver particle is 1.4 times higher than the FRET efficiency assisted by the plasmonic nanogap. This difference in efficiency can be attributed to the lower donor Purcell enhancements in the isolated silver particle (structure 3) (see the inset of Figure 6).

## 4 Conclusions

In conclusion, by extending the donor field and controlling the Purcell enhancement in the system, we have demonstrated long-range Förster energy transfer with sustained high efficiency using plasmonic nanostructures. Our measurements show that the FRET range can be extended to over 200 nm while keeping the FRET efficiency above 0.38, achieving an efficiency enhancement factor of ∼10^8^ with respect to the homogeneous environment. Further optimization can be obtained by controlling the position of the donor and acceptor molecules in the nanogap structure, providing a powerful approach to develop novel light sources, light-harvesting systems, and long-range FRET-imaging and -sensing systems.

## Supporting Information Available

Structural characterizations of the investigated plasmonic nanogaps and thickness measurements, details of the photophysics of the investigated structures, and calculated *G*_FRET_ and FRET efficiency maps (PDF)

## Abbreviations

FRET: Förster resonance energy transfer.

## References

[ref1] Poudel A.; Chen X.; Ratner M. A. Enhancement of Resonant Energy Transfer Due to an Evanescent Wave from the Metal. J. Phys. Chem. Lett. 2016, 7 (6), 955–960. 10.1021/acs.jpclett.6b00119. 26913686

[ref2] Sekar R. B.; Periasamy A. Fluorescence Resonance Energy Transfer (FRET) Microscopy Imaging of Live Cell Protein Localizations. J. Cell Biol. 2003, 160 (5), 629–633. 10.1083/jcb.200210140. 12615908 PMC2173363

[ref3] Mirkovic T.; Ostroumov E. E.; Anna J. M.; Van Grondelle R.; Govindjee; Scholes G. D. Light Absorption and Energy Transfer in the Antenna Complexes of Photosynthetic Organisms. Chem. Rev. 2017, 117 (2), 249–293. 10.1021/acs.chemrev.6b00002. 27428615

[ref4] Olejko L.; Bald I. FRET Efficiency and Antenna Effect in Multi-Color DNA Origami-Based Light Harvesting Systems. RSC Adv. 2017, 7 (39), 23924–23934. 10.1039/C7RA02114C.

[ref5] Mehlenbacher R. D.; McDonough T. J.; Grechko M.; Wu M. Y.; Arnold M. S.; Zanni M. T. Energy Transfer Pathways in Semiconducting Carbon Nanotubes Revealed Using Two-Dimensional White-Light Spectroscopy. Nat. Commun. 2015, 6, 6732 10.1038/ncomms7732. 25865487

[ref6] Kuscu M.; Akan O. B. The Internet of Molecular Things Based on FRET. IEEE Internet Things J. 2016, 3 (1), 4–17. 10.1109/JIOT.2015.2439045.

[ref7] Ghataora S.; Smith R. M.; Athanasiou M.; Wang T. Electrically Injected Hybrid Organic/Inorganic III-Nitride White Light-Emitting Diodes with Nonradiative Förster Resonance Energy Transfer. ACS Photonics 2018, 5 (2), 642–647. 10.1021/acsphotonics.7b01291.

[ref8] Kumari L.; Kar A. K. Compositional Variation Dependent Colour Tuning and Observation of Förster Resonant Energy Transfer in Cd(1-: X)ZnxS Nanomaterials. New J. Chem. 2020, 44 (3), 870–883. 10.1039/C9NJ05199F.

[ref9] Liu L.; He F.; Yu Y.; Wang Y. Application of FRET Biosensors in Mechanobiology and Mechanopharmacological Screening. Front. Bioeng. Biotechnol. 2020, 8, 595497 10.3389/fbioe.2020.595497. 33240867 PMC7680962

[ref10] Ansari A. A.; Thakur V. K.; Chen G. Functionalized Upconversion Nanoparticles: New Strategy towards FRET-Based Luminescence Bio-Sensing. Coord. Chem. Rev. 2021, 436, 213821 10.1016/j.ccr.2021.213821.

[ref11] Forster T. Zwischenmolekulare Energiewanderung Und Fluoreszenz. Ann. Phys. 1948, 437, 55 10.1002/andp.19484370105.

[ref12] Dung H. T.; Knöll L.; Welsch D. G. Intermolecular Energy Transfer in the Presence of Dispersing and Absorbing Media. Phys. Rev. A: At., Mol., Opt. Phys. 2002, 65 (4), 438131–4381313. 10.1103/PhysRevA.65.043813.

[ref13] Gonzaga-Galeana J. A.; Zurita-Sánchez J. R. A Revisitation of the Förster Energy Transfer near a Metallic Spherical Nanoparticle: (1) Efficiency Enhancement or Reduction? (2) the Control of the Förster Radius of the Unbounded Medium. (3) the Impact of the Local Density of States. J. Chem. Phys. 2013, 139, 244302 10.1063/1.4847875. 24387365

[ref14] Novotny L.; Hecht B. Principles of Nano-Optics; Cambridge University Press: Cambridge, 2012. 10.1017/CBO9780511794193.

[ref15] Boddeti A. K.; Guan J.; Sentz T.; Juarez X.; Newman W.; Cortes C.; Odom T. W.; Jacob Z. Long-Range Dipole-Dipole Interactions in a Plasmonic Lattice. Nano Lett. 2022, 22 (1), 22–28. 10.1021/acs.nanolett.1c02835. 34672615

[ref16] Andrew P.; Barnes W. L. Energy Transfer Across a Metal Film Mediated by Surface Plasmon Polaritons. Science 2004, 306 (November), 1002–1005. 10.1126/science.1102992. 15528438

[ref17] Weeraddana D.; Premaratne M.; Gunapala S. D.; Andrews D. L. Controlling Resonance Energy Transfer in Nanostructure Emitters by Positioning near a Mirror. J. Chem. Phys. 2017, 147 (7), 074117 10.1063/1.4998459. 28830167

[ref18] Wubs M.; Vos W. L. Förster Resonance Energy Transfer Rate in Any Dielectric Nanophotonic Medium with Weak Dispersion. New J. Phys. 2016, 18, 053037 10.1088/1367-2630/18/5/053037.

[ref19] Blum C.; Zijlstra N.; Lagendijk A.; Wubs M.; Mosk A. P.; Subramaniam V.; Vos W. L. Nanophotonic Control of the Förster Resonance Energy Transfer Efficiency. Phys. Rev. Lett. 2012, 109 (20), 203601 10.1103/PhysRevLett.109.203601. 23215487

[ref20] Hamza A. O.; Viscomi F. N.; Bouillard J. S. G.; Adawi A. M. Förster Resonance Energy Transfer and the Local Optical Density of States in Plasmonic Nanogaps. J. Phys. Chem. Lett. 2021, 12 (5), 1507–1513. 10.1021/acs.jpclett.0c03702. 33534597

[ref21] Bidault S.; Devilez A.; Ghenuche P.; Stout B.; Bonod N.; Wenger J. Competition between Förster Resonance Energy Transfer and Donor Photodynamics in Plasmonic Dimer Nanoantennas. ACS Photonics 2016, 3 (5), 895–903. 10.1021/acsphotonics.6b00148.

[ref22] De Torres J.; Ferrand P.; Colas Des Francs G.; Wenger J. Coupling Emitters and Silver Nanowires to Achieve Long-Range Plasmon-Mediated Fluorescence Energy Transfer. ACS Nano 2016, 10 (4), 3968–3976. 10.1021/acsnano.6b00287. 27019008

[ref23] Collison R.; Pérez-Sánchez J. B.; Du M.; Trevino J.; Yuen-Zhou J.; O’Brien S.; Menon V. M. Purcell Effect of Plasmonic Surface Lattice Resonances and Its Influence on Energy Transfer. ACS Photonics 2021, 8 (8), 2211–2219. 10.1021/acsphotonics.1c00616.

[ref24] Hamza A. O.; Bouillard J.-S. G.; Adawi A. M. Förster Resonance Energy Transfer Rate and Efficiency in Plasmonic Nanopatch Antennas. ChemPhotoChem 2022, 6 (5), e202100285 10.1002/cptc.202100285.

[ref25] Baibakov M.; Patra S.; Claude J. B.; Wenger J. Long-Range Single-Molecule Förster Resonance Energy Transfer between Alexa Dyes in Zero-Mode Waveguides. ACS Omega 2020, 5 (12), 6947–6955. 10.1021/acsomega.0c00322. 32258931 PMC7114734

[ref26] De Torres J.; Ghenuche P.; Moparthi S. B.; Grigoriev V.; Wenger J. FRET Enhancement in Aluminum Zero-Mode Waveguides. ChemPhysChem 2015, 16 (4), 782–788. 10.1002/cphc.201402651. 25640052

[ref27] Golmakaniyoon S.; Hernandez-Martinez P. L.; Demir H. V.; Sun X. W. Cascaded Plasmon-Plasmon Coupling Mediated Energy Transfer across Stratified Metal-Dielectric Nanostructures. Sci. Rep. 2016, 6 (August), 34086 10.1038/srep34086. 27698422 PMC5048420

[ref28] Bouchet D.; Cao D.; Carminati R.; De Wilde Y.; Krachmalnicoff V. Long-Range Plasmon-Assisted Energy Transfer between Fluorescent Emitters. Phys. Rev. Lett. 2016, 116 (3), 037401 10.1103/PhysRevLett.116.037401. 26849613

[ref29] Akulov K.; Bochman D.; Golombek A.; Schwartz T. Long-Distance Resonant Energy Transfer Mediated by Hybrid Plasmonic-Photonic Modes. J. Phys. Chem. C 2018, 122 (28), 15853–15860. 10.1021/acs.jpcc.8b03030.

[ref30] Lunz M.; Gerard V. A.; Gun’Ko Y. K.; Lesnyak V.; Gaponik N.; Susha A. S.; Rogach A. L.; Bradley A. L. Surface Plasmon Enhanced Energy Transfer between Donor and Acceptor CdTe Nanocrystal Quantum Dot Monolayers. Nano Lett. 2011, 11 (8), 3341–3345. 10.1021/nl201714y. 21755927

[ref31] Murphy G. P.; Gough J. J.; Higgins L. J.; Karanikolas V. D.; Wilson K. M.; Garcia Coindreau J. A.; Zubialevich V. Z.; Parbrook P. J.; Bradley A. L. Ag Colloids and Arrays for Plasmonic Non-Radiative Energy Transfer from Quantum Dots to a Quantum Well. Nanotechnology 2017, 28 (11), 115401 10.1088/1361-6528/aa5b67. 28140370

[ref32] Higgins L. J.; Marocico C. A.; Karanikolas V. D.; Bell A. P.; Gough J. J.; Murphy G. P.; Parbrook P. J.; Bradley A. L. Influence of Plasmonic Array Geometry on Energy Transfer from a Quantum Well to a Quantum Dot Layer. Nanoscale 2016, 8 (42), 18170–18179. 10.1039/C6NR05990B. 27740658

[ref33] Cortes C. L.; Jacob Z. Super-Coulombic Atom-Atom Interactions in Hyperbolic Media. Nat. Commun. 2017, 8, 14144 10.1038/ncomms14144. 28120826 PMC5288497

[ref34] Biehs S. A.; Menon V. M.; Agarwal G. S. Long-Range Dipole-Dipole Interaction and Anomalous Förster Energy Transfer across a Hyperbolic Metamaterial. Phys. Rev. B 2016, 93 (24), 245439 10.1103/PhysRevB.93.245439.

[ref35] Newman W. D.; Cortes C. L.; Afshar A.; Cadien K.; Meldrum A.; Fedosejevs R.; Jacob Z. Observation of Long-Range Dipole-Dipole Interactions in Hyperbolic Metamaterials. Sci. Adv. 2018, 4 (10), eaar5278 10.1126/sciadv.aar5278. 30310865 PMC6173528

[ref36] Marocico C. A.; Zhang X.; Bradley A. L. A Theoretical Investigation of the Influence of Gold Nanosphere Size on the Decay and Energy Transfer Rates and Efficiencies of Quantum Emitters. J. Chem. Phys. 2016, 144 (2), 024108 10.1063/1.4939206. 26772555

[ref37] Karanikolas V.; Marocico C. A.; Bradley A. L. Spontaneous Emission and Energy Transfer Rates near a Coated Metallic Cylinder. Phys. Rev. A: At., Mol., Opt. Phys. 2014, 89 (6), 063817 10.1103/PhysRevA.89.063817.

[ref38] Steele J. M.; Ramnarace C. M.; Farner W. R. Controlling FRET Enhancement Using Plasmon Modes on Gold Nanogratings. J. Phys. Chem. C 2017, 121 (40), 22353–22360. 10.1021/acs.jpcc.7b07317.

[ref39] Yu Y. C.; Liu J. M.; Jin C. J.; Wang X. H. Plasmon-Mediated Resonance Energy Transfer by Metallic Nanorods. Nanoscale Res. Lett. 2013, 8 (1), 209 10.1186/1556-276X-8-209. 23641862 PMC3653766

[ref40] Jackson D. Classical Electrodynamics, 3rd ed.; Wiley, 1998.

[ref41] Lezhennikova K.; Rustomji K.; Kuhlmey B. T.; Antonakakis T.; Jomin P.; Glybovski S.; de Sterke C. M.; Wenger J.; Abdeddaim R.; Enoch S. Experimental Evidence of Förster Energy Transfer Enhancement in the near Field through Engineered Metamaterial Surface Waves. Commun. Phys. 2023, 6 (1), 229 10.1038/s42005-023-01347-1.

[ref42] Pagnotto D.; Muravitskaya A.; Benoit D. M.; Bouillard J.-S. G.; Adawi A. M. Stark Effect Control of the Scattering Properties of Plasmonic Nanogaps Containing an Organic Semiconductor. ACS Appl. Opt. Mater. 2023, 1 (1), 500–506. 10.1021/acsaom.2c00135.

[ref43] Marshall A. R. L.; Roberts M.; Gierschner J.; Bouillard J.-S. G.; Adawi A. M. Probing the Molecular Orientation of a Single Conjugated Polymer via Nanogap SERS. ACS Appl. Polym. Mater. 2019, 1 (5), 1175–1180. 10.1021/acsapm.9b00180.

[ref44] Kumbhar A. S.; Kinnan M. K.; Chumanov G. Multipole Plasmon Resonances of Submicron Silver Particles. J. Am. Chem. Soc. 2005, 127 (36), 12444–12445. 10.1021/ja053242d. 16144364

[ref45] Marshall A. R. L.; Stokes J.; Viscomi F. N.; Proctor J. E.; Gierschner J.; Bouillard J. S. G.; Adawi A. M. Determining Molecular Orientation: Via Single Molecule SERS in a Plasmonic Nano-Gap. Nanoscale 2017, 9 (44), 17415–17421. 10.1039/C7NR05107G. 29104980

[ref46] Edwards A. P.; Adawi A. M. Plasmonic Nanogaps for Broadband and Large Spontaneous Emission Rate Enhancement. J. Appl. Phys. 2014, 115 (5), 053101 10.1063/1.4864018.

[ref47] Sorger V. J.; Pholchai N.; Cubukcu E.; Oulton R. F.; Kolchin P.; Borschel C.; Gnauck M.; Ronning C.; Zhang X. Strongly Enhanced Molecular Fluorescence inside a Nanoscale Waveguide Gap. Nano Lett. 2011, 11 (11), 4907–4911. 10.1021/nl202825s. 21978206

[ref48] Russell K. J.; Liu T.; Cui S.; Hu E. L. Large Spontaneous Emission Enhancement in Plasmonic Nanocavities. Nat. Photonics 2012, 6 (July), 459–462. 10.1038/nphoton.2012.112.

[ref49] Rose A.; Hoang T. B.; McGuire F.; Mock J. J.; Ciracì C.; Smith D. R.; Mikkelsen M. H. Control of Radiative Processes Using Tunable Plasmonic Nanopatch Antennas. Nano Lett. 2014, 14 (8), 4797–4802. 10.1021/nl501976f. 25020029

[ref50] Johnson P. B.; Christy R. W. Optical Constants of the Noble Metals. Phys. Rev. B 1972, 6 (12), 4370–4379. 10.1103/PhysRevB.6.4370.

