# Supplementary material for: Long-Range and High-Efficiency Plasmon-Assisted Förster Resonance Energy Transfer
Source: J Phys Chem C Nanomater Interfaces. 2023 Oct 27;127(44):21611–6. doi: 10.1021/acs.jpcc.3c04281 (PMC10641858; doi:10.1021/acs.jpcc.3c04281)
Supplement: Supplementary file 1 — jp3c04281_si_001.pdf [file jp3c04281_si_001.pdf]

Supporting Information

Long-Range and High Efficiency Plasmonic Assisted Förster Resonance Energy Transfer

Abdullah O. Hamza,[a,b,c*] Ali Al-Dulaimi[a,b,d], Jean-Sebastien G. Bouillard[a,b*] and Ali M. Adawi[a,b*]

a. Department of Physics, University of Hull, Cottingham road, Hull, HU6 7RX, UK. E-mail: a.adawi@hull.ac.uk, j.bouillard@hull.ac.uk and abdulla.hamza@su.edu.krd

b. G. W. Gray Centre for Advanced Materials, University of Hull, Cottingham road, Hull, HU6 7RX, UK.

c. Department of Physics, College of Science, Salahaddin University-Erbil, Erbil, Kurdistan Region, Zipcode 44002, Iraq.

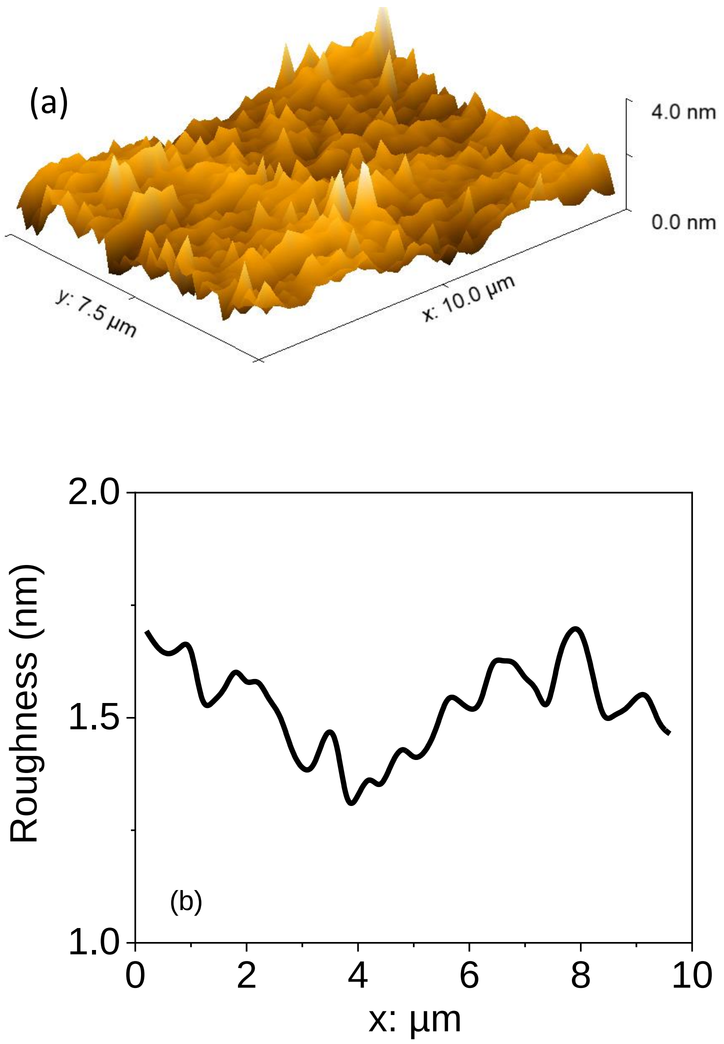

Figure S1: AFM image of the surface of the plasmonic nanogap(a). Averaged roughness along y-axis as a function of x (b).

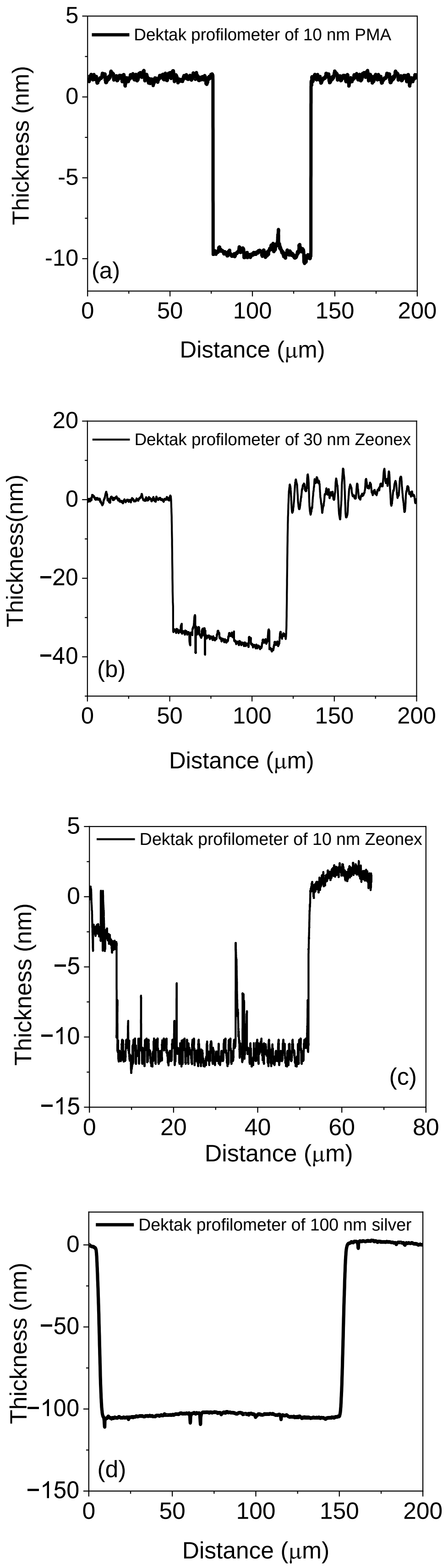

Figure S2: Dektak profilometer of 10 nm of PMA layer (a), 30 nm Zeonex layer (b), 10 nm Zeonex (c) and 100 nm silver (d).

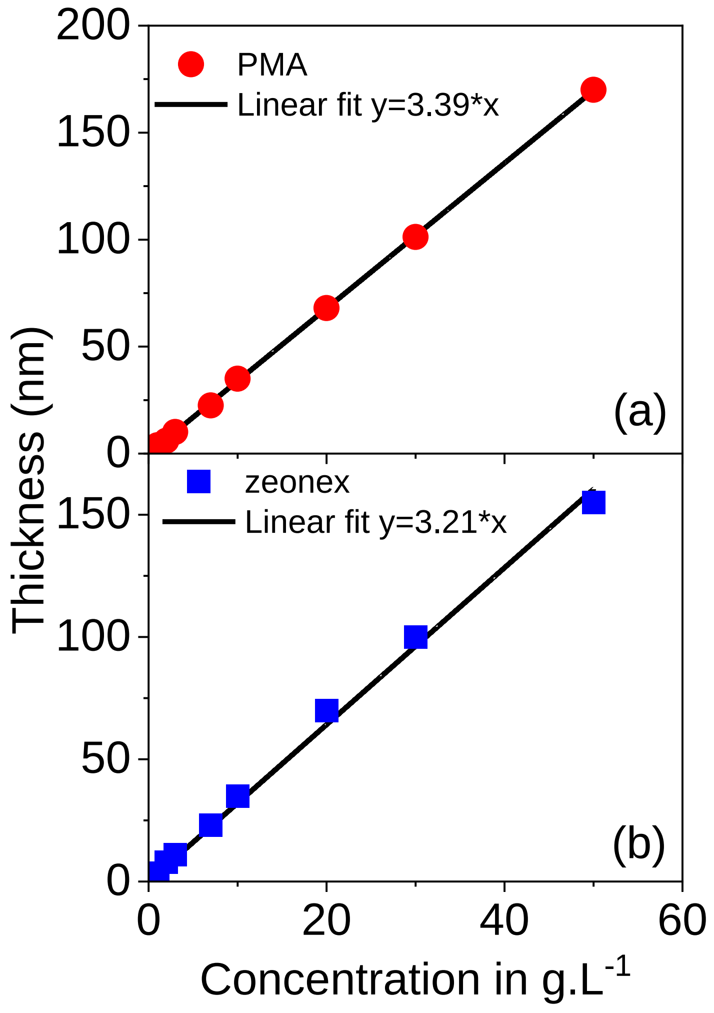

Figure S3: PMA (a) and Zeonex (b) film thickness as a function of concentration.

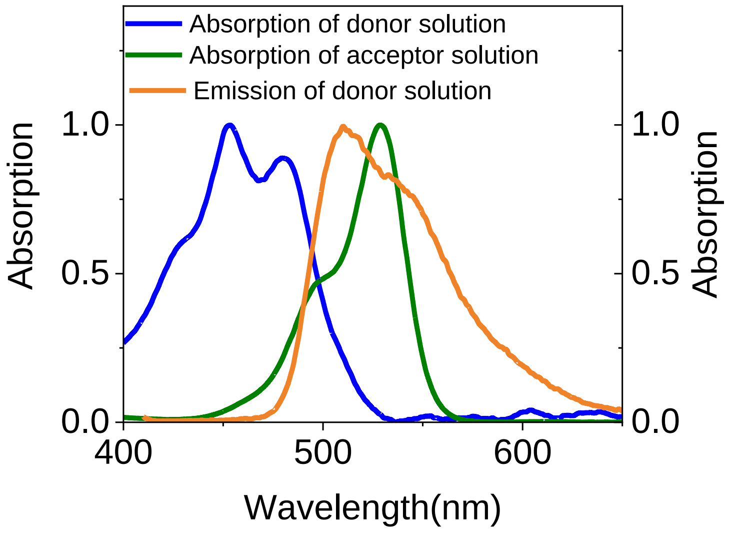

Figure S4: Normalised absorption and emission spectral of the donor Disodium Fluorescein in ethanol solution of PMA alongside the absorption spectrum of acceptor Rhodamine 6G in ethanol solution of PMA. Emission was measured using 405 nm excitation wavelength.

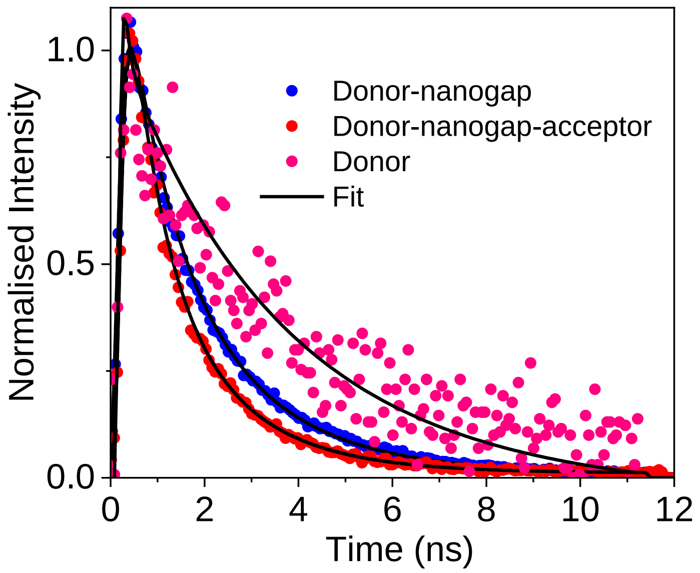

Figure S5: Full fluorescence decay curves of donor-nanogap, donor-nanogap-acceptor measured from a 50 nm plasmonic nanogap with a silver particle of diameter 200 nm. For comparison data are shown for donor film on glass. The donor emission lifetime was extracted via the deconvolution of the instrument response function (IRF) then fitted to double-exponential model: $I(t) = a_1 e^{-\frac{t}{t_1}} + a_2 e^{-\frac{t}{t_2}}$. The fitting parameters are summarised in Table S1.

Table S1: Fitting parameters of figure S5 (Figure 3b)					
Donor-nanogap-acceptor					
Parameters	a_1%	t_1 (ps)	a_2%	t_2 (ps)	χ^2
Average	58	700	42	1788	1.01
Donor only -nanogap					
Parameters	a_1%	t_1 (ps)	a_2%	t_2 (ps)	χ^2
Average	64.08	1138	35.92	2600	1.2
donor only on glass					
Parameters	a_1%	t_1 (ps)	a_2%	t_2 (ps)	χ^2
Average	73	2860	27	4500	0.84

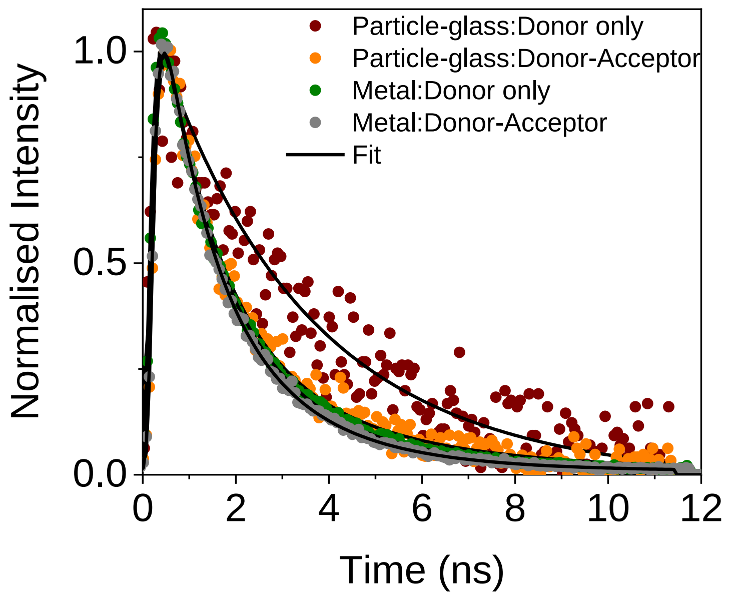

Figure S6: Full fluorescence decay curves of glass-donor-200 nm silver particle, glass-donor-acceptor-200 nm silver particle, metal-donor and metal-donor-acceptor structures. The donor emission lifetime was extracted via the deconvolution of the instrument response function (IRF) then fitted to double-exponential model: $I(t) = a_1 e^{-\frac{t}{t_1}} + a_2 e^{-\frac{t}{t_2}}$. The fitting parameters are summarised in Table S2.

Table S2: Fitting parameters of figure S6 (Figure 4)					
Metal-donor-Acceptor					
Parameters	$a_1\%$	t_1 (ps)	$a_2\%$	t_2 (ps)	χ^2
	76	1100	24	2491	1.01
Particle-glass: donor-acceptor					
Parameters	$a_1\%$	t_1 (ps)	$a_2\%$	t_2 (ps)	χ^2
Average	82	1250	18	4166	1.085
Metal donor only					
Parameters	$a_1\%$	t_1 (ps)	$a_2\%$	t_2 (ps)	χ^2
	78.39	1300	21.61	3000	1.03
Particle-glass donor only					
Parameters	$a_1\%$	t_1 (ps)	$a_2\%$	t_2 (ps)	χ^2
	74.48	2800	25.52	5000	0.95

Table S3: Averaged Γ_D and Γ_{total} with their uncertainties. These values correspond to the average of measurements from 5 identical structures.				
Structure	Γ_D	$\Delta\Gamma_D$	Γ_{total}	$\Delta\Gamma_{total}$
Nanogap	0.879	0.008	1.431	0.027
Metal	0.770	0.012	0.912	0.025
Particle on glass	0.357	0.004	0.802	0.022

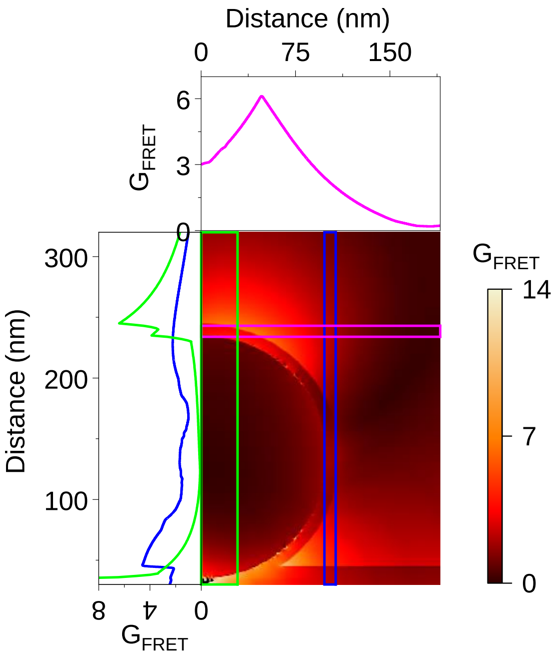

Figure S7: (a) Calculated G_{FRET} map for donor-nanogap-acceptor structure of gap width 50 nm and a silver particle of diameter 200 nm at the donor emission wavelength λ_{donor} = 516 nm averaged over the donor location, with few cross sections showing the variation of G_{FRET} over the acceptor film.

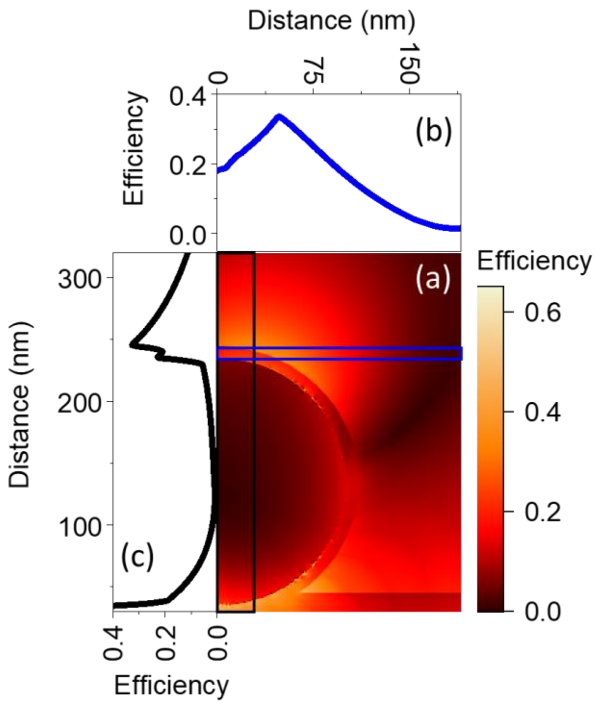

Figure S8: (a) Calculated FRET efficiency map for donor-nanogap-acceptor structure of gap width 50 nm and a silver particle of diameter 200 nm at the donor emission wavelength $\lambda_{donor} = 516$ nm averaged over the donor location. (b) FRET efficiency as a function of x. (c) FRET efficiency as a function of z.

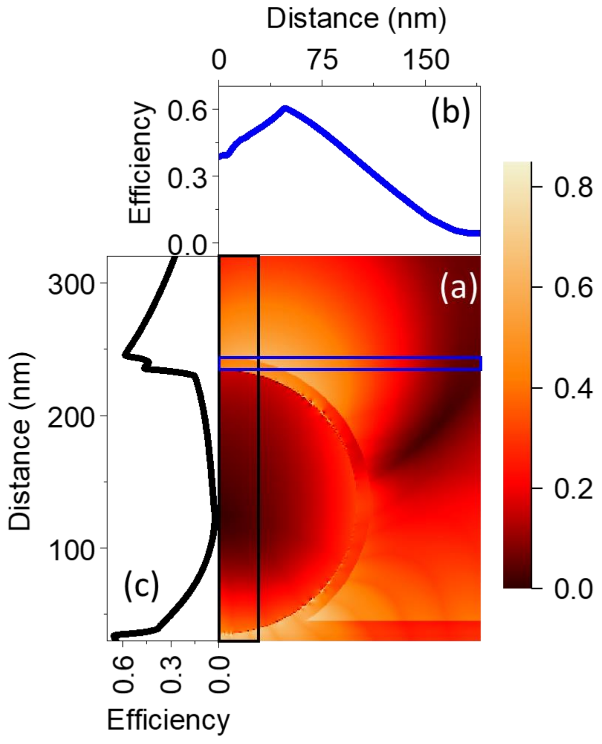

Figure S9: (a) Calculated FRET efficiency map for donor-nanogap-acceptor structure of gap width 50 nm and a silver particle of diameter 200 nm at the donor emission wavelength λ_{donor} = 516 nm, averaged over the donor location and using averaged Purcell factor. (b) FRET efficiency as a function of x. (c) FRET efficiency as a function of z.
